# Inhibitory effects of dietary antioxidants on the formation of carcinogenic polycyclic aromatic hydrocarbons in grilled pork

**DOI:** 10.5713/ajas.18.0805

**Published:** 2019-02-07

**Authors:** Wanwisa Wongmaneepratip, Kriskamol Na Jom, Kanithaporn Vangnai

**Affiliations:** 1 Department of Food Science and Technology, Faculty of Agro-Industry, Kasetsart University, Bangkok 10900, Thailand

**Keywords:** Polycyclic Aromatic Hydrocarbons (PAHs), Grilled Pork, Marinade, Antioxidant, Quercetin, Diallyl Disulfide

## Abstract

**Objective:**

The inhibitory effects of dietary antioxidants, diallyl disulfide (DADS) and quercetin, in marinade were investigated on the formation of carcinogenic polycyclic aromatic hydrocarbons (EPA priority 16 PAHs) in grilled pork.

**Methods:**

The formation of PAHs in grilled sirloin pork with different marinades after charcoal-grilling for 2 min/side were evaluated using high performance liquid chromatography with a photodiode array detector (HPLC-DAD).

**Results:**

Compared with the control marinade treatment (without antioxidant), the addition of DADS (500 mg/kg meat sample) in marinade significantly decreased benzo[*a*]pyrene (B*a*P) (100%) and heavy PAHs (84%) in charcoal-grilled pork, while the addition of quercetin at the same concentration could reduce 23% and 55% of B*a*P and heavy PAHs, respectively.

**Conclusion:**

The results of this study suggested that the addition of DADS in the marinade could be important in decreasing the levels of PAHs in grilled meat.

## INTRODUCTION

Polycyclic aromatic hydrocarbons (PAHs) that form mainly as a result of pyrolysis process, especially the incomplete combustion of organic compounds are a diversified classes of carcinogenic chemicals [1]. These compounds are hydrophobic molecules where the solubility in water decreases as the molecular weight increases and easily dissolve in oil phase [2]. PAHs composed of two to four fused aromatic rings are called light PAHs and those containing more than four aromatic rings are known as heavy PAHs. The heavy PAHs are more stable and more toxic than the light ones [3]. As PAHs represent an important class of carcinogens, PAHs are not toxic but their metabolites that are converted from hydrophobic compounds into relatively hydrophilic compounds during detoxification by the organism are the cause of DNA damage [4]. The International Agency for Research on Cancer [5] has determined that benzo[*a*]pyrene (B*a*P) is carcinogenic to humans (group 1), whereas benz[*a*]anthracene (B*a*A), chrysene (Chry), benzo[*k*]fluoranthene (B*k*F) and benzo[*b*]fluoranthene (B*b*F) are possibly carcinogenic to humans (group 2B). B*a*P is the most widely known and studied of the PAHs due to its importance as one of the most potent animal carcinogenic PAHs, however, the European Food Safety Authority [6] suggested that the sum content of the four PAHs (PAH4: B*a*P, B*a*A, B*b*F and Chry) is a more suitable marker than only B*a*P. European Union (EU) Regulation No 835/2011 stipulated that the maximum level of B*a*P was 2.0 μg/kg and the sum of PAH4 was set at 12.0 μg/kg in smoked meats and smoked meat products [7]. Moreover, PAH16 (Table 1) are classified as priority pollutants by Environmental Protection Agency (EPA) based on carcinogenicity and their occurrence in contaminated foods and the environment is of concern [8]. The definite mechanism of the formation of PAHs is not exactly proven; however, previous researches have proposed that they might be formed through a complicated mechanism (free radical reactions, intramolecular addition, or the polymerization) of small molecules [9] and intramolecular cyclization of lipid peroxides [10]. Various antioxidant compounds have shown effective inhibitory effects on PAH formation in a meat model system [10] and in different types of meat [11,12].

The occurrence of PAHs in food is mainly due to processing at high temperature especially in the charcoal-grilling process because charcoal contains many hydrocarbon compounds which are activated via incomplete combustion to form PAHs [13]. The PAH contamination levels in grilled meats depends on many factors such as types of heating source, grilling time, distance from the heating source, amount of fat, and marinade ingredients [14]. Marinades often contain a lot of additives, oil, herbs and spices to improve the sensory properties (texture, color, etc.) of meat products [15]. Various spices especially garlic and onion are added in the marinade to contribute the unique flavor and taste. Some studies have shown that the addition of ingredients with an antioxidant activity (garlic, onion, lemon juice, etc.) could reduce PAH levels and other carcinogens such as heterocyclic aromatic amines (HCAs) in meat products [16]. The addition of garlic (5 g/kg meat) and onion (65 mL/kg meat) into a marinade could reduce the sum of PAHs in grilled beef from 74.0 μg/kg to 45.2 μg/kg, compared to marinade without the addition of antioxidant [17]. Garlic contains an abundance of chemical compounds that have been shown to possess high antioxidative compounds [18]. Diallyl disulfide (DADS) is the predominant oil-soluble organosulfides in essential garlic oil which has demonstrated an inhibition effect on carcinogenic compounds in meat model system [19]. Quercetin is a flavonoid compound, which is widely contained in onion, tomato, etc. and has been shown to be a strong antioxidant. Quercetin is one of the most powerful scavengers of reactive oxygen species which cause many diseases such as cancers [20].

The reduction of PAH levels in grilled meat by adding ingredients which have antioxidant activities were confirmed on the basis of previous research, however, studying the inhibition effects of pure dietary antioxidants would give a better understanding. Thus, the main objective of this study was to illustrate the effect of the addition two dietary antioxidants, DADS and quercetin, that represent the antioxidant compounds in garlic and onion, respectively in marinade treatments on PAHs formation in charcoal-grilled pork. Our results could provide a theoretical basis for the use of these compounds as potential inhibitors of the PAH formation.

## MATERIALS AND METHODS

### Standards and reagents

PAH16 standards as listed in Table 1 were purchased from Supelco (Bellefonte, PA, USA). HPLC-grade solvents (acetonitrile and dichloromethane) and analytical-grade solvents/chemical (methanol, 2-propanol, 1-buthanol, n-hexane and potassium hydroxide) were purchased from RCI Labscan (Bangkok, Thailand) and Ajax Finechem (Silverwater, NSW, Australia). The DADS and quercetin hydrate were obtained from Sigma-Aldrich (St. Louis, MO, USA).

### Sample preparations

Sirloin pork and all marinade ingredients were obtained from a grocery store in Bangkok, Thailand. Two pieces of 0.5-cm thickness sirloin pork were randomly chosen and immersed in each marinade treatment at 4°±2°C for 1 h. Control marinade (C) (without added pure antioxidant) was composed of 50 g water, 50 g sugar, 20 g oyster sauce, 10 g salt and 7.5 g spice powder per 1 kg meat. DADS and quercetin were added into different marinade treatments: 100 mg/kg diallyl disulfide marinade (D-100), 500 mg/kg diallyl disulfide marinade (D-500), 100 mg/kg quercetin marinade (Q-100), and 500 mg/kg quercetin marinade (Q-500). The marinated samples were charcoal-grilled 2 min/side. The heating source including 500 g of charcoal and 60 g of wood was placed at the bottom of the grill and changed for each sample. The grilled samples were cooled to reach room temperature and packed in aluminum foil bags until used for PAH extraction.

### Extraction and clean up

Sample extraction and clean-up procedures followed our previous study [9]. Meat samples (10 g) were ground and saponified with 100 mL of 2 mol/L of potassium hydroxide in methanol/ water (80:20, v/v) and then extracted with 50 mL of *n*-hexane. This extraction procedure was repeated four times, the collected hexane layers were evaporated using a parallel evaporator at 60°C under reduced pressure. The residue was dissolved in 3 mL of acetonitrile, transferred to an activated Sep-Pak Florisil cartridge (6 mL/1,000 mg, Macherey-Nagel, Langerwehe, Germany) and purged to dryness. Acetonitrile was added to the residue to a final volume of 400 μL and the mixture was subjected to high-performance liquid chromatography-photodiode array detector (HPLC-DAD) analysis. Each sample were conducted in triplicate.

### HPLC-DAD analysis of PAHs

PAH analysis was performed by HPLC-DAD (Waters, Milford, MA, USA) and followed our previous study [9]. Chromatographic resolution was achieved using a reverse phase C18 column (ZORBAX Hypersil ODS column of 250 mm×4.6 mm, 5 μm particle size). PAHs were detected at 254 nm and confirmation by comparing the retention time and DAD spectra (scanned wavelength from 200 to 600 nm) with reference standards. PAHs were quantified using an external standard method. The concentration of each PAH was calculated from its respective calibration curve which obtained by plotting the peak area against the standards at concentration ranged between 0.1 to 20.0 μg/mL (the correlation coefficient ranged from 0.994 to 0.999). The limit of detection (LOD) and quantification (LOQ) of PAHs were determined using signal-to-noise of *S*/*N* = 3 and *S*/*N* = 10 of the lowest concentration of reference standards, respectively [17].

### Statistical analysis

Experiments was performed by a completely randomized design. All experiments were carried out at least in triplicate. Data analysis was processed using analysis of variance, Duncan’s multiple range test and t-test using the SPSS 10.0 software (SPSS, Chicago, IL, USA) to determine whether differences between mean values were significant (p<0.05).

## RESULTS AND DISCUSSION

### Limit of detection, limit of quantification, and recovery study

The LOD and LOQ of PAH16 standards were observed using HPLC-DAD with detection performed at 254 nm and the results ranged from 0.57 to 7.29 μg/L and 1.91 to 22.10 μg/L, respectively. These results showed enough sensitivity for the detection of the compounds in the samples. According to extraction and clean-up method in this study, the recoveries (in percent) of PAHs were in the range of 67.9% to 112.1%, with a mean recovery of 98.5% which satisfactory for determinations at the applied detection level (mg/kg). Some PAHs (Nap, D*ah*A, and Anl) produced a low recovery level because they may have undergone partial loss during clean-up procedure using SPE cartridge [21]. However, based on the AOAC manual guidelines, these recovery values were acceptable [22].

### Inhibitory effect antioxidants to marinade treatments on the formation of PAHs in grilled pork

The contents of PAH compounds in grilled sirloin pork with five different marinade treatments (C, D-100, D-500, Q-100, and Q-500) are shown in Table 2. The results illustrated that the highest concentrations of PAH4 (318.5 μg/kg) and PAH16 (1,173.4 μg/kg) were observed in grilled sirloin pork without the addition of antioxidant in marinade treatment (C) while the samples that treated with all antioxidant marinade treatments (D-100, D-500, Q-100, and Q-500) had lower PAH16 concentrations (878.5, 249.4, 504.4, and 508.5 μg/kg, respectively). Our result was agreed with the complete reduction (100% reduction) of B*a*A and B*ghi*P that was found in grilled sirloin pork treated with D-500, Q-100, and Q-500 marinade treatments compared to control. The definite mechanism of PAHs formation in food is not well understood; however, our results assented with the study reported by Min et al [10] who proposed that the major mechanism of PAHs formation might be formed through free radical reactions. Therefore, our result confirmed that the antioxidant compounds that have free radical scavenging activity could inhibit PAHs formation during charcoal grilling process.

Considering the level of addition antioxidants, Q-100 marinade treatment exhibited significantly lower concentration of light PAHs (438.0 μg/kg) than D-100 marinade treatment (823.6 μg/kg). However, a greater effect of reduction for heavy PAHs was presented by D-100 marinade treatment. Furthermore, grilled sirloin pork treated with D-500 marinade treatment contained the lowest concentrations in both light PAHs and heavy PAHs (223.1 μg/kg and 26.3 μg/kg, respectively) compared to Q-500 marinade treatment (433.4 μg/kg and 75.1 μg/kg, respectively). The results demonstrated that two levels of addition DADS (100 and 500 mg of antioxidant per 1 kg meat sample) in this experiment resulted in a greater reduction of heavy PAHs than that of quercetin at the same concentration. This may have been due to the lipophilic nature of PAHs, DADS that are oil soluble antioxidants (easily dissolved in oil phase similar to PAHs) have a stronger effect on reduction of B*a*P and heavy PAHs in grilled sirloin pork than does quercetin which contains hydroxyl groups and is a more polar antioxidant. Moreover, DADS were reported to be a powerful terminator of lipid peroxidation [18]. DADS was also found to inhibit oxidation reaction by means of radical scavenging but were not involved in chain-braking antioxidant mechanisms [23]. In addition, a comparative computational modeling and analysis of transition state mechanism has suggested that organosulfur compounds derived from garlic with the existence of a thioallyl group, i.e. DADS, play an important role of scavenging ^•^OH and ROO^•^ [24]. Based on its protective effects against free radicals, DADS can be proposed to be involved with the inhibition of PAH formation.

**Figure 1:**
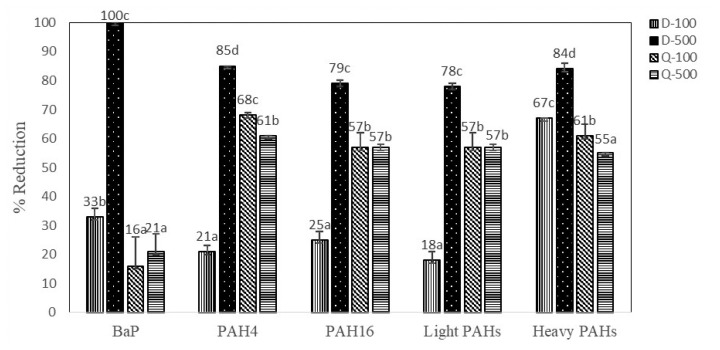
Percentage of reduction of B*a*P, PAH4, PAH16, light and heavy PAHs in grilled sirloin pork treated with different marinade treatments. PAH, polycyclic aromatic hydrocarbon; D-100, 100 mg/kg diallyl disulfide marinated meat sample; D-500, 500 mg/kg diallyl disulfide marinated meat sample; Q-100, 100 mg/kg quercetin marinated meat sample; Q-500, 500 mg/kg quercetin marinated meat sample. ^a,b,c,d^ Mean values in a group followed by the same superscript letter are not significantly different (p>0.05).

Antioxidant marinade treatments (D-100, D-500, Q-100, and Q-500) illustrated inhibitory effects on PAH formation during charcoal grilling process of marinated sirloin pork in comparison to control (C). Figure 1 presents the percentage of reduction of B*a*P as a marker for PAHs contamination, PAH4, PAH16, light PAHs, and heavy PAHs. D-500 marinade treatment showed the highest effect on reducing the generation of B*a*P (100%), PAH4 (85%), PAH16 (79%), light PAHs (78%), and heavy PAHs (84%) in grilled sirloin pork. Reduction of B*a*P level was the highest when the sirloin pork treated with D-500 marinade treatment (100%), followed by D-100 marinade treatment (33%), Q-500 marinade treatment (21%), and Q-100 marinade treatment (16%). According to this experiment, D-500 marinade treatment effectively reduced individual light and heavy PAHs as well as sum of PAH contamination levels (PAH4 and PAH16) in grilled sirloin pork.

In this study, the addition of DADS and quercetin in marinade could reduce the PAH levels in charcoal-grilled pork. Due to the lipophilic property and the presence of thioallyl group, the addition of DADS significantly decreased the PAH concentrations lower than did quercetin in the grilled sirloin pork, especially B*a*P which is considered as carcinogenic to humans (group 1) and heavy PAHs that are more stable and more toxic. As previously mentioned, DADS is antioxidant which naturally present in garlic and onion and has been recognized as part of healthful diet throughout history. DADS is noted in many scientific publications as being associated with medicinal properties and health benefits such as anti-cardiovascular disease, anti-neurological disease, anti-liver disease effects as well as effects for prevention of cold, flu, and arthritis [25]. Importantly, DADS could be a prospective agent for multi-targeted prevention and/or treatment against human cancers because DADS has no toxic effects in healthy cells [26]. Although the adverse effects of very high dose of DADS has been documented, which includes a burning sensation in the mouth and gastrointestinal tract, nausea, diarrhea, vomiting and body odor; in fact, due to the limiting effect of its strong flavor and malodor, it is nearly impossible to add DADS to food at the level that causes adverse effects [26]. In summary, we successfully demonstrated the effectiveness of DADS and quercetin in the inhibition of PAH formation. Our results provide a theoretical basis for the use of these compounds as potential inhibitors of the PAH formation in grilled meat which could be applied to household cooking and the food industry.

## Figures and Tables

**Table 1:** PAH16 compounds defined by Environmental Protection Agency and National Institute of Standards and Technology measured in this study

Compounds	Abbreviation	Structure	Number of rings	Molecular weight*	IARC**
Naphthalene	Nap	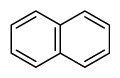	2 (Light PAHs)	128.2	2B
Acenaphthylene	Anl	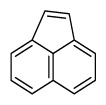	3 (Light PAHs)	152.2	-
Acenaphthene	Ane	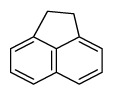	3 (Light PAHs)	154.2	3
Fluorene	Flu	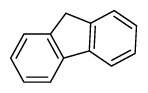	3 (Light PAHs)	166.2	3
Phenanthrene	Phen	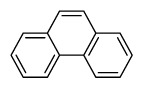	3 (Light PAHs)	178.2	3
Anthracene	Ant	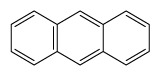	3 (Light PAHs)	178.2	3
Fluoranthene	Flt	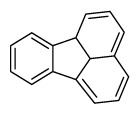	4 (Light PAHs)	202.2	3
Pyrene	Pyr	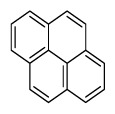	4 (Light PAHs)	202.3	3
Benz[a]anthracene	B*a*A	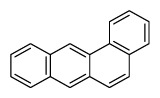	4 (Light PAHs)	228.3	2B
Chrysene	Chry	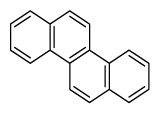	4 (Light PAHs)	228.3	2B
Benzo[*b*]fluoranthene	B*b*F	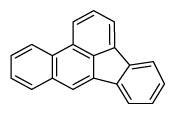	5 (Heavy PAHs)	252.3	2B
Benzo[*k*]fluoranthene	B*k*F	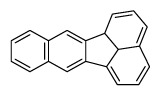	5 (Heavy PAHs)	252.3	2B
Benzo[*a*]pyrene	B*a*P	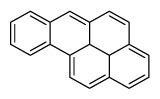	5 (Heavy PAHs)	252.3	1
Dibenz[*a,h*]anthracene	D*ah*A	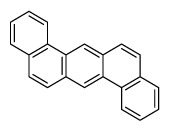	5 (Heavy PAHs)	278.4	2A
Benzo[*g,h,i*]perylene	B*ghi*P	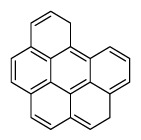	6 (Heavy PAHs)	276.3	3
Indeno[*1,2,3-cd*]pyrene	InP	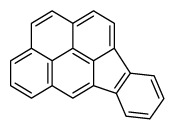	6 (Heavy PAHs)	276.3	2B

PAH16, polycyclic aromatic hydrocarbon 16.

* Bojes and Pope [8].

** IARC [5].

**Table 2:** PAH concentrations (μg/kg) and number of times difference for marinated meat samples compared with control marinated meat sample

PAHs	Concentration1) (μg/kg)				

	C	D-100	D-500	Q-100	Q-500
Light PAHs					
Nap	ND	ND	ND	ND	ND
Anl	ND	ND	ND	ND	ND
Ane	ND	ND	ND	ND	ND
Flu	ND	ND	ND	ND	ND
Phen	171.4±12.5^c^	154.2±7.8^c^ (10)*	68.3±8.4^a^ (60)*	116.5±28.9^b^ (32)*	95.4±17.1^ab^ (44)*
Ant	35.1±9.5^b^	38.1±2.0^b^ (−8)*	9.4±1.9^a^ (73)*	18.1±6.8^a^ (48)*	15.7±0.6^a^ (55)*
Flt	215.3±4.7^d^	115.2±1.6^c^ (47)*	42.9±1.3^a^ (80)*	81.1±8.1^b^ (62)*	71.7±16.6^b^ (67)*
Pyr	353.5±37.0^d^	302.6±10.2^c^ (14)*	69.6±11.3^a^ (80)*	168.6±33.3^b^ (52)*	170.8±1.8^b^ (52)*
B*a*A	67.2±2.9^b^	60.7±6.9^b^ (10)*	ND (100)*	ND (100)*	ND (100)*
Chry	164.5±9.2^d^	152.8±6.0^d^ (7)*	32.9±5.3^a^ (80)*	53.7±7.4^b^ (67)*	79.8±5.6^c^ (52)*
Total light PAHs	1,007.0±75.8^d^	823.6±34.5^c^	223.1±28.2^a^	438.0±84.5^b^	433.4±41.7^b^
Heavy PAHs					
B*b*F	56.6±15.3^b^	18.1±4.6^a^ (68)*	16.2±0.8^a^ (71)*	21.9±5.2^a^ (61)*	21.1±4.7^a^ (63)*
B*k*F	18.9±3.1^ab^	16.8±2.7^a^ (11)*	10.1±0.9^a^ (47)*	19.4±11.7^ab^ (−3)*	30.4±5.9^b^ (−60)*
B*a*P	30.2±4.1^d^	20.0±1.7^b^ (34)*	ND (100)*	25.1±0.3^c^ (18)*	23.6±1.5^bc^ (23)*
D*ah*A	ND	ND	ND	ND	ND
B*ghi*P	60.7±4.8^b^	ND (100)*	ND (100)*	ND (100)*	ND (100)*
InP	ND	ND	ND	ND	ND
Total heavy PAHs	166.4±27.3^c^	54.9±9.0^ab^	26.3±1.7^a^	66.4±17.2^b^	75.1±12.1^b^
PAH42)	318.5±31.5^d^	251.6±19.2^c^	49.1±6.1^a^	100.7±12.9^b^	124.5±11.8^b^
PAH16	1,173.4±103.1^d^	878.5±43.5^c^	249.4±29.9^a^	504.4±101.7^b^	508.5±53.8^b^

PAH, polycyclic aromatic hydrocarbon; ND, not detected; B*a*A, benz[*a*]anthracene; B*b*F, benzo[*b*]fluoranthene; B*k*F, benzo[*k*]fluoranthene; B*a*P, benzo[*a*]pyrene.

1) C, control marinade treatment; D-100, 100 mg/kg diallyl disulfide marinade treatment; D-500, 500 mg/kg diallyl disulfide marinade treatment; Q-100, 100 mg/kg quercetin marinade treatment; Q-500, 500 mg/kg quercetin marinade treatment.

2) PAH4: B*a*A, Chry, B*b*F, and B*a*P.

* Percentage of reduction compared with control marinated meat sample.

a–d Mean values in a row followed by the same lowercase superscript letter are not significantly different (p>0.05).
